# Feedback for Promoting Motor Skill Learning in Physical Education: A Trial Sequential Meta-Analysis

**DOI:** 10.3390/ijerph192215361

**Published:** 2022-11-21

**Authors:** Yankun Han, Syed Kamaruzaman Bin Syed Ali, Lifu Ji

**Affiliations:** 1 Faculty of Education, University of Malaya, Kuala Lumpur 50603, Malaysia; 2 College of Physical Education, Jilin Normal University, Siping 136000, China

**Keywords:** feedback, motor skill learning, physical education, students, meta-analysis, TSA

## Abstract

Feedback can be used as an effective teaching method in physical education (PE) to promote students’ learning of motor skills. However, there is no objective synthetic evidence to support the role of feedback in PE. Additionally, the effect of each feedback subtype on students’ motor skill learning is still unclear. This study aimed to conduct a meta-analysis and trial sequential analysis (TSA) to evaluate the effects of feedback and feedback subtypes on students’ motor skill learning. Nine databases were searched through September 2022 to identify appropriate literature. Meta-analysis was conducted using Review Manager 5.4 software and TSA was performed using TSA version 0.9.5.10 beta software. Fifteen studies were included. Feedback significantly improved students’ motor skill learning in PE (SMD 0.47; 95% CI 0.01, 0.93; *Z* = 2.02; *p* = 0.04). The TSA confirmed the result of the meta-analysis. Sensitivity analyses showed that the subtypes of feedback, including visual feedback, visual combined verbal feedback, visual self-model, visual expert model, corrective feedback, and teacher-regulated feedback, significantly improved students’ learning of motor skills. In contrast, verbal, evaluative, and informational feedback did not produce changes in motor skill learning. Both complex and simple motor skills were improved by feedback. The use of feedback in PE benefits motor skill learning, regardless of whether the motor skills are complex or simple.

## 1. Introduction

Countries around the world consider the development of students’ motor skills as one of the main common goals of school physical education, such as the United States [1], China [2], and Malaysia [3]. Students’ degree of mastery of motor skills can be defined as motor competence [4], a relatively permanent change in the ability to perform motor skills [5]. It has been reported that the development of motor competence is positively related to participation in physical activity (PA) and moderate to vigorous physical activity (MVPA) in children and adolescents [6]. Children and adolescents with more developed motor skills are more actively involved in PA and MVPA than their peers with poorer motor skills [7,8,9]. Motor competence developed in childhood is one of the potential key factors for promoting motor skills and PA in adolescence [6,10]. The favorable relationship between motor competence and PA is critical for child and adolescent health, especially for reducing sedentary behavior [9,11] and preventing obesity [7]. Considering the great potential benefits of learning motor skills for students’ health-related fitness, school PE needs to use effective teaching techniques to develop students’ motor competence.

The learning of motor skills is usually based on the theory of information processing [12]. Feedback as an external source of information facilitates changes in motor performance that reflect learning [12]. Therefore, feedback as information is a crucial factor in learning motor skills. [13]. Feedback is the process that realizes the parameterization of action representation [14]. Learners can use cognition to process the temporal and spatial information of actions into symbols, which can then be represented in the brain and modified and extracted as needed [15,16]. In the teaching of PE, students need the information to explore the parameters of movements and then achieve the effect of correcting and learning motor skills. However, previous studies have provided conflicting findings on the influence of feedback on students’ motor skill learning. Some studies suggest that feedback can effectively promote students’ motor skill learning [17,18,19,20]. In contrast, some studies in recent years have not found positive evidence that feedback can promote motor skill learning in PE lessons, such as volleyball [21], shot-put [22], and standing long jump [23]. The reason for the conflicting results of different studies might be the selection and application of subtypes of feedback, such as form (verbal feedback or visual feedback), content (corrective feedback, evaluative feedback, or informative feedback), and schedule (self-controlled feedback or regulated feedback) [5,24]. Although recent systematic reviews have reported the effect of feedback on students’ motor skill learning, the reported evidence for subtypes of feedback on motor skill learning is inconsistent [14,24,25]. Therefore, it is necessary to further clarify the effects of each feedback subtype on students’ motor skill learning.

Due to the limited inclusion criteria, none of the existing systematic reviews has yet conducted a meta-analysis on the effect of feedback on the promotion of motor skill learning in school PE [14,24,25]. Whereas, a meta-analysis is considered the best reliable evidence due to the power and precision of the estimated intervention effect [26,27,28]. Therefore, this study aims to conduct a meta-analysis to investigate the effects of feedback and feedback subtypes on students’ motor skill learning in PE lessons. Based on the experience from existing systematic reviews [14,24,25] and the feedback characteristics used in the included articles, this study defines feedback used in PE as follows: Teachers of PE provide information in various forms and contents for students’ motor performance to promote students’ learning of motor skills in the lessons of PE and then divided them into three subtypes of feedback: Feedback Form, Feedback Content, and Feedback Schedule. In addition, we conducted a trial sequential analysis (TSA) to control for false positives (type I errors) that result from a cumulative meta-analysis to make the estimated results more conservative. The results of this meta-analysis provide more objective data on the effect of feedback in promoting students’ learning of motor skills in PE and clarify the role of each feedback subtype in motor skill learning. Furthermore, the results provide objective evidence for the use of feedback as an effective instructional technique in PE.

## 2. Methods

This meta-analysis was performed according to the PRISMA guidelines (Preferred Reporting Items for Systematic Reviews and Meta-Analysis) [29].

### 2.1. Eligibility Criteria

Inclusion criteria: (1) Feedback was used as an intervention technique in general school physical education (PE). (2) The research must use feedback as the main variable and test its effectiveness on changes in students’ motor skill levels. (3) The study was designed as a pretest and posttest with a quantitative approach reflecting causality between feedback (independent variable) and motor skill learning (dependent variable). Primary feedback was used as an intervention technique in the experimental groups, regardless of form or content. Control groups were non-feedback or other secondary feedback. (4) Included articles must be published, peer-reviewed articles in English. Exclusion criteria: (1) Research background was outside the natural setting of school PE and participants were not regular students. (2) Students with disabilities. (3) Studies that did not investigate feedback-induced changes in motor skill learning. (4) Gray literature, including dissertations, theses, reviews, conference proceedings, and unpublished articles.

### 2.2. Search Strategy

A systematic literature search of nine databases was conducted to identify relevant published articles addressing the topic of feedback to improve motor skill learning in PE. The various search terms used were (1) “feedback” OR “augmented feedback” OR “external feedback” OR “extrinsic feedback” OR “feedback frequency” OR “visual feedback” OR “verbal feedback” OR “knowledge of result” OR “knowledge of performance” OR “KR” OR “KP” (2) “motor skill” OR “sports skill” OR “athletic skill” OR “moment skill” OR “motor performance” OR “motor learning” OR “skill learning” OR “skill acquisition” OR “skill training” (3) “physical education” OR “school sports” OR “PE” OR “student*” OR “college*” OR “universit*” OR “school” OR “class*” OR “lesson*” OR “curricul*” OR “instruct*”. The timeline for searching potentially eligible articles was through September 2022. The databases and strategies used for the search were listed in Table 1. Reference lists of included studies and relevant review articles from recent years were also manually searched for potentially suitable articles.

### 2.3. Data Extraction

Data from each included study were extracted using a standard form. Study characteristics included the following information: (1) First author and year of publication; (2) participant characteristics, including sample type, sample size, mean age, and initial ability level; (3) motor skills; (4) task complexity; (5) duration of intervention; (6) feedback format; (7) feedback content; (8) feedback schedule; (9) main results.

### 2.4. Risk of Bias Assessment

The risk of bias in the included studies was assessed using the Cochrane Risk of Bias Tool [30]. Ratings were classified as high, unclear, and low based on bias of selection, performance, detection, attrition, and reporting. The study with a low risk of bias in all items was rated as a high-quality study.

### 2.5. Quality of Evidence Assessment

The quality of evidence was assessed using the GRADE (Grading of Recommendations, Assessment, Development, and Evaluations). This grading system was divided into four quality levels: Very low, low, moderate, and high. We used the GRADEpro Guideline Development Tool (GRADEpro GDT) to assess the quality of evidence based on five items, namely risk of bias, inconsistency, indirectness, imprecision, and publication bias.

### 2.6. Reliability of the Selection of Included Studies

Two research members (YK and LF) performed the selection of studies separately. Disagreements between the two members were resolved through discussion or, if necessary, the third research member (SA) was consulted to reach a consensus.

### 2.7. Statistical Analysis

Considering the possible contamination of the pretest intervention measure on the posttest, the mean difference (MD) and the pooled standard deviation (SD) were used for the meta-analysis [31]. We calculated the MD and the pooled SD based on the following formulas: MD = posttest mean − pretest mean, pooled SD = (√[(SD_1_^2^ + SD_2_^2^)/2]) [31], mean = (first quartile + median + third quartile)/3, and SD = (third quartile − first quartile)/1.35 [32].

We conducted meta-analysis using Review Manager 5.4 software (The Cochrane Collaboration, 2020). For continuous variables, MD was reported with 95% confidence interval (CI). The inverse variance was used to assess effect size [33]. Due to the different measurement scales in the included studies, the combined effect size was estimated based on the standardized mean difference (SMD); otherwise, MD was used for identical measurement indicators [30]. The *I*^2^ statistic was applied to report heterogeneity, where *I*^2^ ≥ 50% indicates the presence of heterogeneity [30]. We performed subgroup analysis to determine the source of heterogeneity. The random effects model was used when *I*^2^ > 30% to reduce the influence of variation [27]. The fixed effects model was used when *I*^2^ ≤ 30% [27]. Moreover, we visually inspected the funnel plot to assess publication bias. A *p* < 0.05 represented statistical significance for analyses.

Trial sequential analysis (TSA) was then applied to verify the primary result. Although meta-analysis is considered the most reliable evidence due to the higher power and precision of the estimated intervention effects [26,27,28], false positives (type I errors) may be reported due to the increased risk of systematic and random bias [26,27] from sparse data and repeated testing [28,34]. TSA, a method that provides the required information size in a cumulative meta-analysis [27], can reduce false significant results by adjusting the *p*-value and expanding the confidence interval based on the sample size to obtain statistical significance [26]. According to the TSA User’s Guide (https://ctu.dk/tsa/ (accessed on 11 October 2022)), a sufficient level of evidence for the expected intervention effect is achieved when the cumulative *Z*-curve exceeds both the traditional boundary (*Z* = 1.96) and the trial sequential monitoring boundary. On the contrary, if the cumulative *Z*-curve exceeds neither the traditional boundary (*Z* = 1.96) nor the trial sequential monitoring boundary, and does not reach the required information size, it indicates a lack of evidence to draw a conclusion. We performed TSA by setting the type I error (*α*) and power (1 − *β*) at 5% and 80%, respectively. All monitoring boundary was defined as two-sided. TSA estimation was responsible for calculating the required information size. The DerSimonian and Laird random effects model was applied [33] using TSA version 0.9.5.10 beta software to run TSA (https://ctu.dk/tsa/ (accessed on 11 October 2022)).

Primary outcome analysis was performed on all included studies. For the primary outcome data (mean ± SD), we considered only the main indicator that reflected the purpose of the study, which was to assess the effect of feedback on students’ motor skill learning in PE. When two or more similar indicators emerged, research members in the current study discussed and agreed on the indicator that best-represented motor skills. Sensitivity analyses for the primary outcome were conducted based on the following subgroups: Visual feedback, verbal feedback, visual combined verbal feedback, visual self-model, visual expert model, evaluative feedback, corrective feedback, informative feedback, teacher-regulated feedback schedule, complex motor skills, and simple motor skills.

## 3. Results

### 3.1. Studies Selection

**Figure 1:**
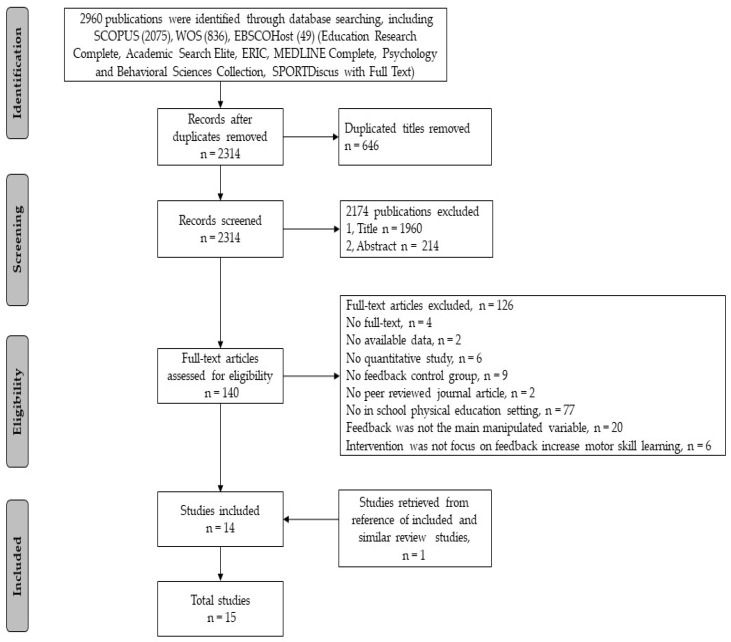
PRISMA flowchart of study selection.

The process of study selection is shown in Figure 1. A total of 2960 articles were identified using predefined electronic databases. After removing duplicate titles, titles, and abstracts of articles that did not meet the inclusion criteria, the remaining 140 articles were selected for full-text review. Furthermore, we excluded 126 studies according to the eligibility criteria. One article was included after a manual search of the reference lists of 14 included studies and review articles on related topics. Finally, 15 articles were eligible for this trial sequential meta-analysis [17,18,19,20,21,35,36,37,38,39,40,41,42,43,44].

### 3.2. Study Characteristics

Table 2 shows the main characteristics of the 15 articles included in this trial sequential meta-analysis. The publication period ranged from 1970 to 2021, and the number of articles from the last 10 years was eight [17,18,19,20,41,42,43,44], accounting for 53% of the total. The participants of the study ranged from elementary school to college, and most of the students were beginners in their motor skills. Different motor skills were used as instructional content, including eight studies with complex tasks [19,20,21,36,37,42,43,44], four studies with simple tasks [17,35,40,41], and three studies that combined complex and simple tasks [18,38,39]. The forms of feedback used in the research were mainly visual and verbal feedback. The content of feedback included correction, information, and evaluation. The feedback schedule was mainly regulated by the PE teacher.

### 3.3. Risk of Bias Assessment

**Figure 2:**
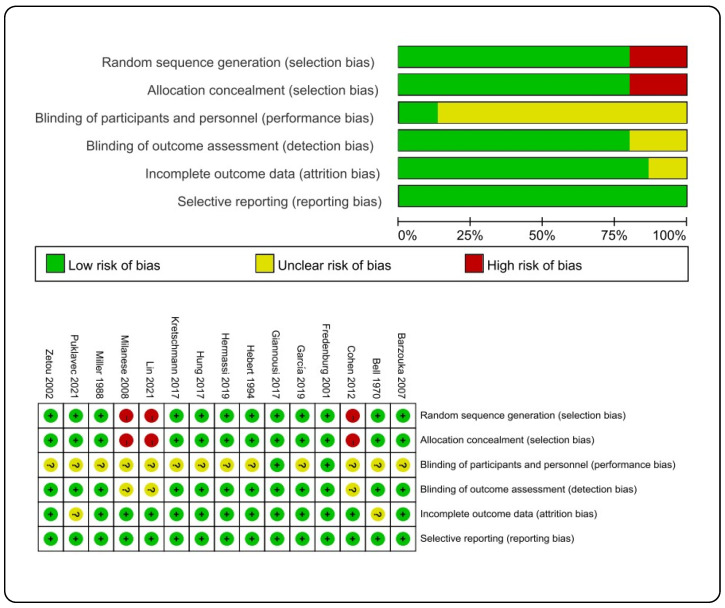
Risk of bias assessment. Zetou (2002) [39], Puklavec (2021) [20], Miller (1988) [36], Milanese (2008) [40], Lin (2021) [19], Kretschmann (2017) [44], Hung (2017) [43], Hermassi (2019) [18], Hebert (1994) [37], Giannousi (2017) [42], García (2019) [17], Fredenburg (2001) [38], Cohen (2012) [41], Bell (1970) [35], Barzouka (2007) [21].

The risk of bias is shown in Figure 2. Twelve studies reported random sequence generation [17,18,20,21,35,36,37,38,39,42,43,44], and twelve studies used the concealed allocation approach [17,18,20,21,35,36,37,38,39,42,43,44]. Only two studies designed the protocol in order that participants were blinded [38,42]. Twelve studies referred to blinding of outcome assessment [17,18,20,21,35,36,37,38,39,42,43,44]. Two studies were unclear about reporting incomplete outcome data [20,35]. Null study related to selective reporting. Two studies were considered to be of high quality [38,42].

### 3.4. Methodological Quality

The GRADE quality of evidence for all studies, visual feedback, visual combined verbal feedback was high. However, the quality of the visual self-model, visual expert model, corrective feedback, teacher-regulated feedback, complex motor skills, and simple motor skills were moderate. The quality of verbal feedback, evaluative feedback, and information was low. The quality of evidence was decreased mainly due to inconsistency. Table 3 shows the details of the GRADE quality of evidence for the outcomes.

### 3.5. Publication Bias

Visual inspection of the funnel plot of all studies included in the meta-analysis revealed no significant publication bias. Please see Figure S1.

### 3.6. Primary Outcomes

**Figure 3:**
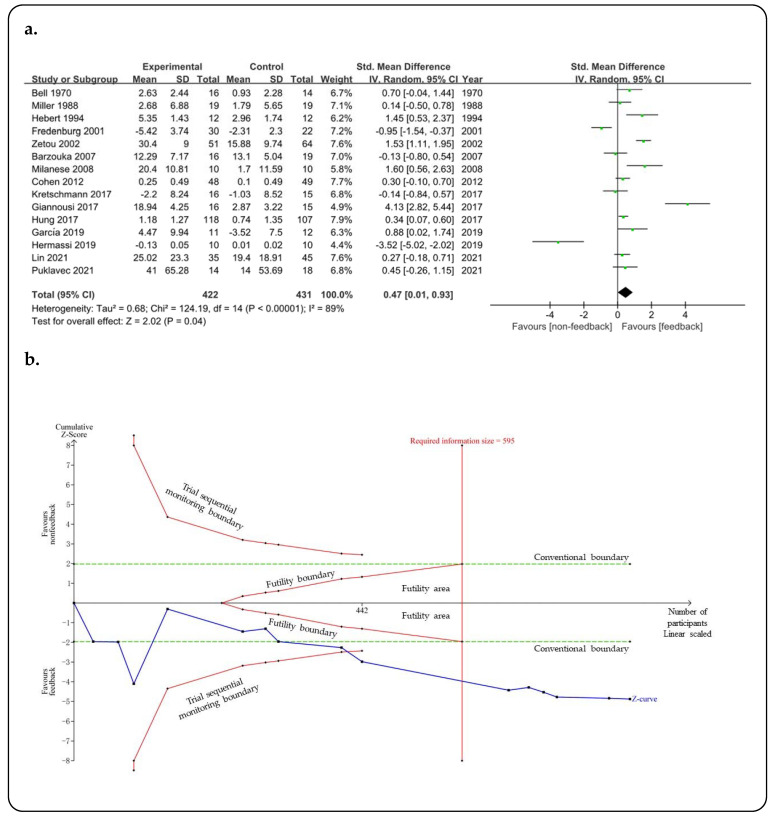
Effect of feedback on motor skill learning across all included studies. (**a**) Forest plot of feedback vs. non-feedback on motor skill learning. Bell (1970) [35], Miller (1988) [36], Hebert (1994) [37], Fredenburg (2001) [38], Zetou (2002) [39], Barzouka (2007) [21], Milanese (2008) [40], Cohen (2012) [41], Kretschmann (2017) [44], Giannousi (2017) [42], Hung (2017) [43], García (2019) [17], Hermassi (2019) [18], Lin (2021) [19], Puklavec (2021) [20]. (**b**) Trial sequential analysis to assess the effect of feedback on motor skill learning with two-sided monitoring boundary, *α* = 5%, *β* = 20%. Required information size was calculated based on TSA estimation, which was 595.

All 15 studies with 853 participants included in this trial sequential meta-analysis were used to analyze the effects of feedback on students’ learning of motor skills in PE. As shown in Figure 3a, the SMD was 0.47 (95% CI 0.01, 0.93; *Z* = 2.02; *p* = 0.04) for feedback versus non-feedback. The TSA showed that the *Z*-curve exceeded both the traditional boundary and the trial sequential monitoring boundary and reached the required information size (Figure 3b). Thus, there is sufficient evidence that feedback can significantly enhance the learning of motor skills in PE.

**Figure 4:**
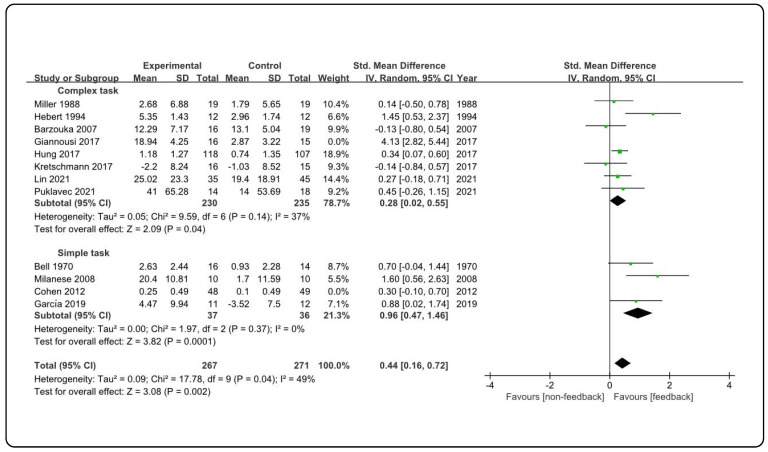
Heterogeneity analysis. The first and second black diamond represents the effect estimate of the meta-analysis of the complex task group and simple task group respectively, and the third black diamond represents the overall effect estimate of the meta-analysis; the green box represents the point estimate of the effect for a single study. Studies for complex task group are Miller (1988) [36], Hebert (1994) [37], Barzouka (2007) [21], Giannousi (2017) [42], Hung (2017) [43], Kretschmann (2017) [44], Lin (2021) [19], Puklavec (2021) [20]; studies for simple task group are Bell (1970) [35], Milanese (2008) [40], Cohen (2012) [41], García (2019) [17].

The stability of the result was further tested by performing sensitivity analyses. As shown in Table 3, in terms of the form of feedback, visual feedback (SMD = 0.71; 95% CI 0.14, 1.28; *Z* = 2.45; *p* = 0.01) and visual combined verbal feedback (SMD = 1.15; 95% CI 0.26, 2.05; *Z* = 2.53; *p* = 0.01) significantly improved students’ motor skills in PE, while verbal feedback (SMD = −0.09; 95% CI −1.01, 0.83; *Z* = 0.20; *p* = 0.84) did not significantly change motor skills. Both the self-model (SMD = 1.15; 95% CI 0.00, 2.29; *Z* = 1.97; *p* = 0.05) and the expert model (SMD = 0.85; 95% CI −0.01, 1.70; *Z* = 1.93; *p* = 0.05) were significantly beneficial for learning motor skills in PE. With regard to the content of feedback, of the three types of content, only corrective feedback (SMD = 1.49; 95% CI −0.02, 3.00; *Z* = 1.94; *p* = 0.05) had a significant effect on motor skill learning. In addition, feedback had a significant effect on learning motor skills of different task complexities, as evidenced by significantly improving both complex motor skills (SMD = 0.62; 95% CI 0.10, 1.13; *Z* = 2.34; *p* = 0.02) and simple motor skills (SMD = 0.73; 95% CI 0.22, 1.24; *Z* = 2.81; *p* = 0.005). The schedule of feedback was mainly regulated by the PE teacher (SMD = 0.74; 95% CI 0.18, 1.30; *Z* = 2.57; *p* = 0.01), which significantly improved the motor skills of the students. Subgroup analysis indicated that heterogeneity could be caused by task complexity (Figure 4).

## 4. Discussion

The results of our meta-analysis suggest that the use of feedback in physical education (PE) can significantly improve the learning of motor skills in students. A subsequent trial sequential analysis (TSA) confirmed this positive evidence. Meanwhile, this conclusion was also supported by most of the sensitivity analysis results.

First, using the meta-analysis and the TSA for the 15 included studies, we found that the use of feedback in PE was more likely to lead to improvements in students’ motor skill learning than the use of non-feedback. This finding was consistent with the conclusion of a recent systematic review [24]. Although a different study design and inclusion criteria were used, the review summarized 23 studies using the best evidence synthesis method and presented strong evidence that feedback was more beneficial than no feedback for improving students’ motor skills in PE. In general, feedback as an external source of information is critical for motor skill learning [13]. This is due to the fact that learners can use cognition to process the temporal and spatial information of actions into symbols, which can then be represented in the brain and modified and extracted as needed [15,16]. Feedback is the process that realizes the parameterization of action representation [14]. In PE, students need the information to explore the parameters of movements and then achieve the effect of correcting and learning motor skills. Therefore, feedback is inevitable and indispensable for students in the natural PE setting. However, the conclusion of some studies did not support the effect of feedback in PE [21,22,45]. The reason for the contradictory results might be that feedback in PE is usually achieved through several different subtypes and combinations of subtypes. For example, different forms of feedback (visual or verbal), feedback content (corrective, evaluative, or informative), and feedback schedule (self-controlled or regulated).

Then, we performed sensitivity analyses on feedback subtypes. The results showed that all feedback subtypes, in addition to verbal feedback, evaluative feedback, and informative feedback, can improve students’ motor skills in PE. According to the characteristics of the feedback used in the 15 included studies, we further classified the feedback form into three groups, namely visual feedback, verbal feedback, and visual combined verbal feedback. The results of the sensitivity analyses showed that, except for verbal feedback, both visual feedback and visual combined verbal feedback were effective in promoting motor skill learning. Moreover, we conducted sensitivity analyses on the stability of visual feedback on motor skill learning with regard to the observation of the expert model and self-model. The results showed that both models in PE can significantly improve students’ motor skills. This finding was supported by evidence from recent systematic reviews [14,25]. Mödinger et al. (2021) systematically reviewed 11 studies and found that the use of video-based visual feedback in PE can effectively improve students’ learning of motor skills in certain school settings [25]. Han et al. (2022) conducted a systematic review of 18 included studies and reported that visual feedback with observational learning as the main strategy can effectively promote students’ motor skill learning in PE, and the conclusion was supported by strong evidence [14]. In addition, Han et al. (2022) stated in their systematic review that in PE, observation of the expert model or self-model for learning motor skills has its advantages [14]. It is recommended that the complexity of the task and the initial skill level of the student be fully and carefully considered to determine which model is more appropriate for learning motor skills [14]. Consistent with the conclusion we found, Mödinger et al. (2021) also reported in a systematic review that the combination of visual and verbal feedback was more effective in promoting students’ learning of motor skills than verbal feedback alone [25]. In contrast, Han et al. (2022) reported conflicting evidence in their systematic review of the effect of combining visual and verbal feedback on motor skill learning [14]. Zhou et al. (2021) also reported in their systematic review that there is limited evidence for the superiority of visual feedback over verbal feedback in facilitating motor learning [24]. In another systematic review of 13 studies, Starzak et al. (2022) indicated that verbal feedback is beneficial for learners when learning complex gymnastics techniques [46].

Theoretically, verbal feedback has always been considered an effective instructional strategy to promote motor skill learning in PE [47]. This is due to the fact that verbal feedback can not only enable students to receive effective attentional information when learning motor skills [48], but also compensate for the lack of visual feedback caused by students’ carelessness [49]. The reason for the inconsistent conclusions of different studies may be that the use of verbal feedback in PE is affected by many potential factors, especially the nature of the content of verbal feedback [14]. In the sensitivity analysis of the feedback content dimension, we also found that except for corrective feedback, neither evaluative feedback nor informative feedback had a significant effect on motor skill learning. Zhou et al. (2021) reported conflicting evidence on the effect of feedback content on motor skill learning in a systematic review [24], which supports our findings. It is worth noting that this review study also indicated that task complexity may influence the selection and application of feedback content in PE [24]. Therefore, we conducted a sensitivity analysis for the effect of feedback on complex motor skills and simple motor skills, respectively. The results showed that feedback significantly improved students’ learning of complex and simple motor skills in PE. Our results were partially supported by findings from previous studies. For example, Tzetzis et al. (2008) reported that corrective feedback was more helpful for learning complex motor skills since this type of feedback caused learners to receive supportive information and increased their confidence in learning [50]. Johnson et al. (2001) found that corrective verbal feedback from peers in elementary school PE significantly improved the learning of complex ball striking skills [51]. However, some studies have reported that informative feedback or a combination of information and evaluative feedback was beneficial for middle and elementary school students in learning simple motor skills, such as volleyball [52] and tennis ball overhand throw [41]. Therefore, it can be confirmed that task complexity is indeed a potential factor influencing the selection and application of feedback content. Our subgroup analysis of heterogeneity confirmed this conclusion. The subgroup analysis of task complexity showed that heterogeneity decreased from 89% to 78% and heterogeneity decreased to 49% when one study after another was excluded. The heterogeneity of the two subgroups, the complex motor skills group, and the simple motor skills group, also decreased to 37% and 0%, respectively. This suggests that task complexity resulted in high heterogeneity. Among the studies, Cohen et al. (2012) used corrective feedback when teaching a simple task, which resulted in high heterogeneity in the simple task subgroup [41]. Giannousi et al. (2017) used corrective feedback when teaching complex tasks, which resulted in high heterogeneity in the complex task subgroup [42]. The reason for this contradictory result might be that students’ initial ability level could be another potential factor influencing the selection and application of feedback content [24]. Braun et al. (2017) pointed out that informative feedback is more beneficial for novice motor learners since they are in the cognitive phase of learning and need detailed information to master each movement [53]. This explains why the corrective feedback that Giannousi et al. (2017) [42] used to teach novices complex tasks resulted in high heterogeneity in the complex task subgroup.

With this consideration, 11 studies (11/15, 73%) in this meta-analysis used novices as subjects and 12 studies (12/15, 80%) used regulated verbal feedback from PE teachers. It is suggested that with comprehensive consideration of task complexity and initial student’s ability, the use of teacher-regulated corrective feedback in PE should be an effective instructional strategy to promote motor skill learning. Furthermore, seven of eight visual feedback studies used video-based visual feedback to help students learn motor skills. With the rapid development of science and technology, convenient visual mobile devices are increasingly used by learners and teachers in the field of motor skills. In future teaching, video-based visual feedback can be considered as a regular strategy for PE. Although this meta-analysis served to estimate the effect of each feedback subtype on motor skill learning in a more robust and precise measure, comparing the effect of each subtype on motor skill learning went beyond the purpose of this study. Therefore, it is necessary to carefully explain which subtype of feedback better promotes motor skill learning in PE. It is suggested that many more comparative empirical studies need to be conducted in the future to test the differences in the effects of different combinations of feedback on students’ motor skill learning.

## 5. Limitations

We should note that this study has some limitations. First, two articles that were eligible for inclusion did not have complete reported data, and the data required for the meta-analysis could not be calculated even with the existing data in the articles. We had attempted to contact the corresponding author or first author of the articles by e-mail, but we were unable to obtain a positive response. Therefore, the articles that met the inclusion criteria were missing, which affected the results of the meta-analysis to some extent. However, according to the results of the TSA, this did not affect the effect of feedback on students’ motor skill learning in PE. It is recommended that articles report the mean ± SD and confidence interval at the time of publication, which can truly reflect the degree of concentration and dispersion of the data, to more accurately capture the degree of influence of feedback on motor skill learning. Second, to comply with the procedures of meta-analysis and TSA, we excluded studies that did not include a control group according to the inclusion criteria, thus the current study was not able to compare the effect of including all feedback in the experimental conditions. It is recommended that the inclusion of a control group in the study design be considered to more accurately compare the effects of different feedback on motor skill learning. Third, the main purpose of this study was to investigate the effect of feedback on motor skill learning. Therefore, in the main results, we only compared the effect of feedback and non-feedback, but not the different categories of feedback in form, content, and schedule. As a result, we cannot determine which form, content, or schedule of feedback is more beneficial for motor skill learning in PE. Fourth, the qualifications and educational background of PE teachers could be potential factors influencing the delivery of feedback. Unfortunately, however, the included studies did not explicitly provide information about the PE teachers who participated in instruction. Therefore, it is difficult for this study to assess whether the background of PE teachers has an influence on the delivery of feedback in PE classes. Future research is needed to clarify these issues.

## 6. What Does this Article Add?

As shown in Table S1, several previous systematic reviews on the similar topic have reported on the effect of feedback on students’ motor skill learning in PE. However, this meta-analysis differs from these studies since, first, we used the criteria to include only quantitative studies. Therefore, the results of this study represent the effect of feedback on motor skill learning in PE with more objective data that reduce the subjective judgment. Second, we conducted a meta-analysis. Meta-analysis is currently considered the best available evidence since it increases the power and precision of the estimated intervention effect. Third, to control for false positives (type I errors) resulting from a cumulative meta-analysis, we performed a TSA to make the estimated results more conservative and to confirm the result of the meta-analysis. Finally, sensitivity analyses were conducted to test the stability of the effects of feedback on motor skill learning.

## 7. Conclusions

This trial sequential meta-analysis suggests that the use of feedback in PE can help students learn motor skills, whether they are complex or simple. In terms of the form of feedback, visual feedback and visual combined verbal feedback can significantly improve students’ motor skills, but verbal feedback does not affect the change in motor skills. In the case of visual feedback, observation of both the expert model and the self-model can significantly improve the level of motor skills. As for the content of feedback, corrective feedback can significantly improve students’ motor skills, but evaluative feedback and informative feedback do not influence the change in motor skills. The feedback schedule is mainly based on the schedule regulated by the PE teacher, which can significantly improve students’ motor skills. It is suggested that many more comparative empirical studies should be conducted in the future to investigate the differences in the effects of different feedback combinations on students’ motor skill learning in PE. Based on the above, this study has practical significance for practitioners in the field of PE and training. For example, physical education teacher education (PETE) students, PE teachers, PETE educators and coaches. Practitioners in the field could refer to the findings of this study to incorporate feedback strategies into motor skills instruction for students or athletes. With the rapid development of technology, practitioners could use the cutting-edge visualization devices to incorporate visual feedback elements into instructional or training practices and provide supplemental verbal feedback as needed to compensate for information omitted through visual neglect. With visual feedback, practitioners could choose to display the expert model or the learner’s self-model depending on the actual situation. As for the application of verbal feedback, corrective feedback conveyed by the teacher is an ideal strategy to promote learners’ motor skills. In addition, based on the results of this study, policy makers of the PETE program may refer to the following suggestions to improve the professionalism of PETE students and educators. First, with the rapid development of technology, visualized mobile devices are increasingly popular. The PETE program could consider the skills in applying visualized mobile devices as mandatory content to improve practitioners’ ability to apply the most commonly used video-based visual feedback. Second, the PETE program could consider developing a manual of evaluation criteria for the use of feedback in PE to improve practitioners’ ability to apply feedback in practice. For example, the assessment could simulate real-life situations in PE and assess the examinee’s ability to select and apply different types of feedback at the appropriate time.

## Figures and Tables

**Table 1:** Search strategies.

Database	Outcomes	Search String
Scopus	2075	(TITLE-ABS-KEY(“feedback” OR “augmented feedback” OR “external feedback” OR “extrinsic feedback” OR “feedback frequency” OR “visual feedback” OR “verbal feedback” OR “knowledge of result” OR “knowledge of performance” OR “KR” OR “KP”) AND TITLE-ABS-KEY(“motor skill” OR “sports skill” OR “athletic skill” OR “moment skill” OR “motor performance” OR “motor learning” OR “skill learning” OR “skill acquisition” OR “skill training”) AND TITLE-ABS-KEY(“physical education” OR “school sports” OR “PE” OR “student*” OR “college*” OR “universit*” OR “school” OR “class*” OR “lesson*” OR “curricul*” OR “instruct*”)) AND (LIMIT-TO(DOCTYPE, “ar”)) AND (LIMIT-TO (LANGUAGE, “English”))
Web of Science	836	Results for ((TS = (“feedback” OR “augmented feedback” OR “external feedback” OR “extrinsic feedback” OR “feedback frequency” OR “visual feedback” OR “verbal feedback” OR “knowledge of result” OR “knowledge of performance” OR “KR” OR “KP”)) AND TS = (“motor skill” OR “sports skill” OR “athletic skill” OR “moment skill” OR “motor performance” OR “motor learning” OR “skill learning” OR “skill acquisition” OR “skill training”)) AND TS = (“physical education” OR “school sports” OR “PE” OR “student*” OR “college*” OR “universit*” OR “school” OR “class*” OR “lesson*” OR “curricul*” OR “instruct*”) and Article or Early Access (Document Types) and English (Languages)
EBSCOHost	49	SU (“feedback” OR “augmented feedback” OR “external feedback” OR “extrinsic feedback” OR “feedback frequency” OR “visual feedback” OR “verbal feedback” OR “knowledge of result” OR “knowledge of performance” OR “KR” OR “KP”) AND SU (“motor skill” OR “sports skill” OR “athletic skill” OR “moment skill” OR “motor performance” OR “motor learning” OR “skill learning” OR “skill acquisition” OR “skill training”) AND SU (“physical education” OR “school sports” OR “PE” OR “student*” OR “college*” OR “universit*” OR “school” OR “class*” OR “lesson*” OR “curricul*” OR “instruct*”) in Title, Abstract, Keywords. Limiters: English, Peer Reviewed Academic Journal

Note: EBSCOHost includes Education Research Complete, Academic Search Elite, ERIC, MEDLINE Complete, Psychology, Behavioral Sciences Collection, and SPORTDiscus with Full Text; “*” represents wildcard.

**Table 2:** Summary characteristics of included studies.

Study	Participants				Motor Skills	Task Complexity	Intervention Length	Feedback Format	Feedback Content	Feedback Schedule	Main Results
	Sample Type	Sample Size	Mean Age	Skill Level							
Bell (1970) [35]	University students	30 EG: 16, CG: 14	NA	Novice	Handball toss	Simple	4 weeks	EG: Visual-SM, CG: Verbal	Corrective	NA	1:EG↑, CG→ 2:EG = CG
Miller (1988) [36]	University students	55 EG1: 17, EG2: 19, CG: 19	NA	Novice	Tennis drive	Complex	1200 min	EG1: Visual-SM + verbal, EG2: Visual-EM + verbal, CG: verbal	NA	TR	1: EG1, 2→, CG→ 2: EG1 = EG2 = CG
Hebert (1994) [37]	University students	48 EG1: 12, EG2: 12, EG3: 12, CG: 12	20.92 ± 2.68 y	Novice	Tennis forehand volley	Complex	5 blocks, 50 trails	EG1: Verbal, EG2: Visual, EG3: Verbal + Visual, CG: no	Corrective + evaluative	TR	1: EG3 ↑ > EG1,2↑, 2: EGs > CG
Fredenburg (2001) [38]	Elementary school students	103 EG1: 20, EG2: 30, EG3: 31, CG: 22	NA	Novice	Cup-stacking skills	Simple and complex	4 days	EG1: Verbal-EV, EG2: Verbal-IN, EG3: Verbal-EV+IN, CG: no	Evaluative (EV) + information (IN)	TR	Sample task: EG1,2,3 = CG, complex task: EG2 > EG 3 > EG1 > CG
Zetou (2002) [39]	Elementary school students	116 EG: 52, CG: 64	11.7 ± 0.5 y	Novice	Volleyball serve and set	Simple and complex	8 weeks	EG: Visual-EM + verbal, CG: Visual-SM + verbal	Information	TR	1:EG↑, CG↑ 2:EG > CG
Barzouka (2007) [21]	High school students	53 EG1: 18, EG2: 16 CG: 19	13.1 ± 0.9 y	Novice	Volleyball, reception	Complex	6 weeks	EG1: Visual-EM + verbal, EG2: Visual- SM+EM + verbal, CG: verbal	Corrective	TR	1: EG1, EG2, CG ↑, 2: EG1 = EG2 = CG
Milanese (2008) [40]	High school students	30 EG1: 10, EG2: 10, CG: 10	13 y	NA	Standing long jump	Simple	3 weeks	EG1: Verbal-error, EG2: Verbal-CO, CG: no	Corrective (CO)	TR	1: EG1, EG2,↑, 2: EG1 > EG2, CG
Cohen (2012) [41]	Elementary school students	97 EG: 48, CG: 49	8.78 ± 4.76 y	Novice	Tennis ball overhand throw	Simple	1 week	EG: Verbal-EV+CO, CG: Verbal-EV	Evaluative (EV) + corrective (CO)	TR	1:EG↑, 2:EG > CG
Giannousi (2017) [42]	University students	60 EG1: 15, EG2: 16, EG3: 14, CG: 15	18.7 ± 1.82 y	Novice	Freestyle swimming	Complex	7 weeks	EG1: Visual-SM + verbal, EG2: Visual-EM + verbal, EG3: Verbal, CG: no	Corrective	TR	1: EG1, 2, 3↑, CG→, 2: EG1 > EG2 > EG3 > CG
Hung (2017) [43]	University students	225 EG: 118, CG: 107	NA	NA	Badminton serve, clear	Complex	5 months	EG: Visual, CG: no	NA	NA	1: EG↑, 2: EG > CG
Kretschmann (2017) [44]	Secondary school students	31 EG: 16, CG: 15	NA	Experienced	Swimming front crawl	Complex	7 weeks	EG: Visual-EM, CG: no	Information	TR	1:EG↑, 2:EG > CG
García (2019) [17]	University students	35 EG1: 11, EG2: 12, CG: 12	20.26 ± 2.16 y	Novice	Handball throwing	Simple	3 sets of pitches, total 30	EG1: Verbal- positive, EG2: Verbal-negative, CG: no	Evaluative	TR	1: EG1↑, EG2↓, CG↓, 2:EG1 > EG2, CG
Hermassi (2019) [18]	University students	20 EG: 10, CG: 10	EG: 21.8 ± 0.5 y, CG: 22.1 ± 0.2 y	NA	15, 30 m sprint, T-half, and ZIG-ZAG test	Simple and complex	8 weeks	EG: Verbal CG: no	Evaluative	TR	1:EG↑,CG→, 2:EG > CG
Lin (2021) [19]	University students	144 EG1: 35, CG1: 45, EG2: 34, CG2: 30	19–22 y	Novice	Badminton, EG1: smash, EG2: backhand driving	Complex	8 weeks	EG1,2: Visual-SM+EM, CG1,2: no	NA	NA	1:EG↑,CG→ 2:EG1 > CG1, EG2 > CG2
Puklavec (2021) [20]	Elementary school students	75 EG1: 19, EG2: 24, EG3: 14, CG: 18	11 ± 0.5 y	Novice	Long jump	Complex	8 weeks	EG1: Verbal + key error, EG2: Verbal + visual + key error, EG3: Verbal + visual + all error, CG: no	Information	TR	1:EG3↑, 2:EG3 > EG1, 2, CG

Abbreviations: EG: Experimental group; CG: Control group; y: Years old; NA: Not applied; SM: Self-model; EM: Expert model; TR: Teacher regulated; “↑” increased; “→” no change; “↓” decreased; “>” better than; “=” no difference.

**Table 3:** The GRADE quality of evidence for primary outcome and sensitivity analysis.

Results	Number of Studies	SMD, Random 95% CI	*p*	*Z*	Quality
All studies	15 [17,18,19,20,21,35,36,37,38,39,40,41,42,43,44]	0.47 [0.01, 0.93]	0.04	2.02	High
Visual feedback	8 [19,21,35,36,39,42,43,44]	0.71 [0.14, 1.28]	0.01	2.45	High
Verbal feedback	6 [17,18,20,38,40,41]	−0.09 [−1.01, 0.83]	0.84	0.20	Low
Visual + verbal feedback	6 [20,21,36,37,39,42]	1.15 [0.26, 2.05]	0.01	2.53	High
Visual self-model	4 [19,35,36,42]	1.15 [0.00, 2.29]	0.05	1.97	Moderate
Visual expert model	6 [19,21,36,39,42,44]	0.85 [−0.01, 1.70]	0.05	1.93	Moderate
Evaluative feedback	2 [17,18]	−1.28 [−5.59, 3.04]	0.56	0.58	Low
Corrective feedback	4 [21,35,40,42]	1.49 [−0.02, 3.00]	0.05	1.94	Moderate
Informative feedback	3 [20,39,44]	0.64 [−0.41, 1.70]	0.23	1.19	Low
TR Feedback	12 [17,20,21,35,36,37,38,39,40,41,42,44]	0.74 [0.18, 1.30]	0.01	2.57	Moderate
Complex motor skills	8 [19,20,21,36,37,42,43,44]	0.62 [0.10, 1.13]	0.02	2.34	Moderate
Simple motor skills	4 [17,35,40,41]	0.73 [0.22, 1.24]	0.005	2.81	Moderate

Abbreviations: GRADE: Grading of Recommendations Assessment, Development, and Evaluation; SMD: Std. mean difference; CI: Confidence interval; TR: Teacher regulated.

## Data Availability

Data supporting the conclusions of this article are available from the included studies and are provided by the authors without reservation.

## Supplementary Materials

The following supporting information can be downloaded at: https://www.mdpi.com/article/10.3390/ijerph192215361/s1. Figure S1: Funnel plot for publication bias; Table S1: Comparison with recent systematic reviews on the same topic.
