# Supplementary material for: Feedback for Promoting Motor Skill Learning in Physical Education: A Trial Sequential Meta-Analysis
Source: Int J Environ Res Public Health. 2022 Nov 21;19(22):15361. doi: 10.3390/ijerph192215361 (PMC9690366; doi:10.3390/ijerph192215361)
Supplement: Supplementary file 1 [file ijerph-19-15361-s001.zip › ijerph-2004467-supplementary.pdf]

Table S1. Comparison with Recent Systematic Reviews on Same Topic

Author	Zhou/2021 [24]	Mödinger/2021 [25]	Han/2022 [14]	The Present Study
Meta-analysis	NA	NA	NA	Applied
Search strategy until (month/year)	April 2021	June 2020	June 2022	September 2022
Trial sequential analysis	NA	NA	NA	Applied
GRADE	NA	NA	NA	Applied
Research focus	Feedback format and content	Feedback format	Feedback format and content	Feedback format, content, schedule
VIF on MSL (SMD, 95% CI)	NA	NA	NA	0.71 [0.14, 1.28]
VEF on MSL (SMD, 95% CI)	NA	NA	NA	-0.09 [-1.01, 0.83]
VIF + VEF on MSL (SMD, 95% CI)	NA	NA	NA	1.15 [0.26, 2.05]
VSM on MSL (SMD, 95% CI)	NA	NA	NA	1.15 [0.00, 2.29]
VEM on MSL (SMD, 95% CI)	NA	NA	NA	0.85 [-0.01, 1.70]
EVF on MSL (SMD, 95% CI)	NA	NA	NA	-1.28 [-5.59, 3.04]
COF on MSL (SMD, 95% CI)	NA	NA	NA	1.49 [-0.02, 3.00]
INF on MSL (SMD, 95% CI)	NA	NA	NA	0.64 [-0.41, 1.70]
TRF on MSL (SMD, 95% CI)	NA	NA	NA	0.74 [0.18, 1.30]
CMS on MSL (SMD, 95% CI)	NA	NA	NA	0.62 [0.10, 1.13]
SMS on MSL (SMD, 95% CI)	NA	NA	NA	0.73 [0.22, 1.24]

Abbreviations: NA-not applied; GRADE-Grading of Recommendations Assessment, Development, and Evaluation; SMD-Std. mean difference; CI-confidence interval; MSL-motor skill learning; VIF-visual feedback; VEF-verbal feedback; VIF + VEF-visual combined verbal feedback; VSM-visual self-model; VEM-visual expert model; EVF-evaluative feedback; COF-corrective feedback; INF-informative feedback; TRF-teacher regulated feedback; CMS-complex motor skills; SMS-simple motor skills.

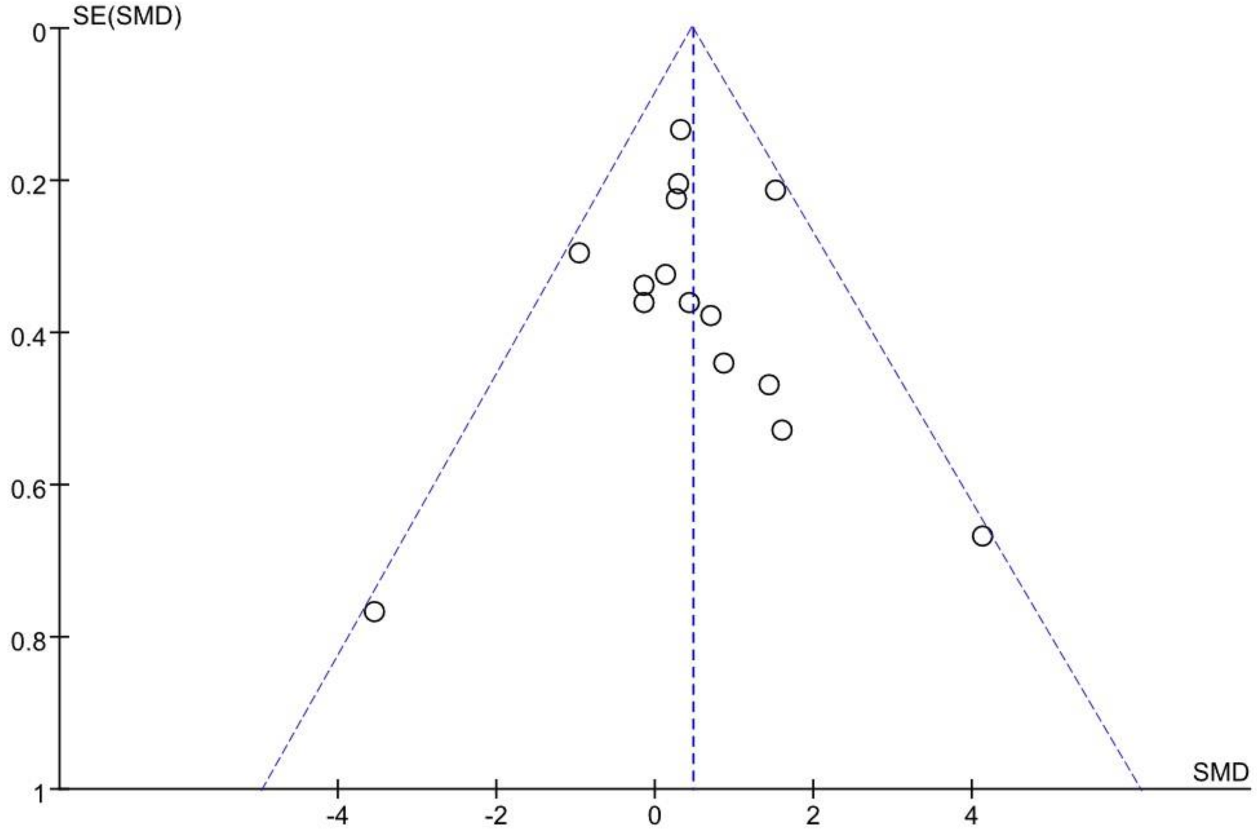

Figure S1. Funnel Plot for Publication Bias
